# Assessing the prices and affordability of oncology medicines for three common cancers within the private sector of South Africa

**DOI:** 10.1186/s12913-021-06627-6

**Published:** 2021-07-06

**Authors:** Phyllis Ocran Mattila, Zaheer-Ud-Din Babar, Fatima Suleman

**Affiliations:** 1 Department of Pharmacy, University of Huddersfield, Queensgate, Huddersfield, HD1 3DH UK; 2 Discipline of Pharmaceutical Sciences, School of Health Sciences, Westville Campus, University of KwaZulu-Natal, Private Bag X54001, Durban, 4000 South Africa

**Keywords:** Oncology, Cancer, Medicine, Pricing, Affordability, South Africa

## Abstract

**Background:**

Prices of cancer medicines are a major contributor to the cost of treatment for cancer patients and the comparison of these cost needs to be assessed.

**Objectives:**

To assess the prices of cancer medicines for the three most common cancers ((breast, prostate and colorectal) in the private healthcare sector of South Africa.

**Methods:**

The methodology was adapted from the World Health Organization (WHO)/ Health Action International (HAI) methodology for measuring medicine prices. The Single Exit Price (SEP) variations between product types of the same medicine between the highest- and lowest-priced product and between Originator Brand (OB) and its Lowest Priced Generic (LPG) of the same medicine brand was compared, as of March 2020. The affordability of those medicines for cancer usage based on treatment affordability in relation to the daily wage of the unskilled Lowest-Paid Government Worker (LPGW) was also determined. Also, a comparison of the proportion of the population below the poverty line (PL) before (I_pre_) and after (I_post_) procurement of the cancer medicines was determined.

**Results:**

SEP Price differences ranged from 25.46 to 97.33% between highest- and lowest-priced products and a price variation of 72.09% more for the OB than the LPG medicine, except for one LPG that was more expensive than the OB. Affordability calculations showed that All OB treatments for all three cancers (breast, prostate and colorectal), except for paclitaxel 300 mg (0.2 days wage) and Fluorouracil (Fluroblastin) 500 mg (0.3 days wage) costs respectively were more than 1 day’s wage, with patients diagnosed with colorectal cancer needing 32.5 days wages in order to afford a standard course of treatment for a month.

**Conclusion:**

There was a considerable variation in the price of different brands of cancer medicines available in the South African private sector.

## Introduction

The global cancer burden is estimated to have risen to 18.1 million new cases and is responsible for an estimated 9.6 million deaths in 2018 [1]. Globally, about 1 in 6 deaths is due to cancer. Unless greater effort is done to alter the course of the disease, this number is expected to rise to close to 30 million new cases by 2040. About 70% of deaths from cancer occur in Low-and Middle-Income countries (LMICs) [1]. In South Africa, the estimated cancer cases are 107,464 in 2018 and may increase to 177,773 in 2040 [2]. In South Africa, breast, prostrate and colo-rectal cancer rank in the top 10 cancers with cases of 15,491, 13,152 and 7354 respectively in 2020 [2, 3]. It is known that early detection and treatment may improve health outcomes associated with the disease for adults [1] and this would depend on the equitable access to available and affordable low cost highly active cancer medicines.

Cancer treatment is expensive and high prices of cancer medicines have a huge impact on access in LMICs. Most of the newer cancer medicines and new therapies such as immunotherapy, monoclonal antibodies, and targeted therapy are out of reach for the large populations with poor socio-economic conditions in LMICs and even the older cytotoxic agents remain only affordable to a minority of patients. For example, according to a World Health Organization report, a course of standard treatment for early-stage human epidermal growth factor receptor 2 positive (HER2+) breast cancer (doxorubicin, cyclophosphamide, docetaxel, trastuzumab) would cost about 10 years of average annual wages in India and South Africa [4]. According to the World Bank (WB), South Africa is regarded as an Upper Middle-Income Country (UMIC) with a population of 59,308,690 (mid-year estimate) [5], in 2020 and a Gross National Income (GNI) per capita of US$ 6040 in 2019 [6].

The World Bank (WB) has defined the international Poverty Line (PL) as US $1.90 per person per day using data on purchasing power parities and an expanded set of household income and expenditure surveys in 2011 [7]. This defines the cost of basic needs in some of the poorest countries in the world and is the absolute minimum threshold for defining poverty. However, for an upper middle-income country like South Africa, the PL has been set at US $5.50 per person per day, thus defining the cost of basic needs in South Africa [8]. If the pre-payment income (income before purchasing the medicine) is above the US$5.50 poverty line and post-payment income (income left after purchasing the medicine) falls below this poverty line then the purchasing of that medicine has impoverished people in South Africa [9].

The World Health Statistics 2020 reported that 1.4% of South Africans spend less than 10% their total household expenditure or income on health [10]. Some of this expenditure is due to public sector–dependent (unemployed or low earning) uninsured persons, accessing health care, including medicines, in the private sector [11]. Government funded healthcare is offered to all South Africans for free, yet people can opt to purchase private insurance in order to be treated at private hospitals and health clinics. Patients in the private sector (generally the wealthy) can either pay for their health care needs via a medical aid scheme (insurance) or the patient is faced with an out-of-pocket expenditure. Out-of-pocket (OOP) expenditure could be related to co-payments for treatment; nutritional changes in diet; rehabilitation, and travel to appointments. The extent of OOP for cancer patients is unknown in South Africa.

South Africa has implemented several important medicine pricing interventions in the post-apartheid era, informed by the 1996 National Drug Policy (NDP) [12]. One key aim of the NDP of South Africa is to promote the availability of safe and effective medicines at the lowest possible cost by monitoring and negotiating medicine prices and by rationalising the medicine pricing system in the public and private sectors and by promoting the use of generic medicines [13, 14].

The Department of Health has adopted measures using regulations to address the pricing of medicines and one of them was the introduction of the SEP [11–14]. The SEP in terms of the regulations means “the price at which the manufacturer or importer of a medicine or scheduled substance can sell to a wholesaler or distributor. It combines the ex-manufacturer price as well as a logistic fee portion. The wholesaler or distributor will then sell the medicine to a pharmacist who adds a dispensing fee before the medicine is sold to a patient.” This is complemented with a provision for a regulated maximum increase in the single exit price, determined annually by the Minister of Health, on the advice of the Pricing Committee [15], and the maximum capped percentage increase varies each year. Manufacturers can take the maximum increase, part of the increase, no increase, or even reduce their prices annually. The introduction of the SEP resulted in an approximately 22% reduction in medicine prices and saved the scheme about ZAR 319 million per year in medicine expenditure since 2004 [16]. In terms of private sector pricing, the SEP mechanism and the publication of annual adjustments has provided the state with a powerful tool [11, 12, 17].

The impact of the SEP on affordability though is unclear. Scarcity of pricing or affordability data is one of the major barriers in the development of effective and transparent pricing policy in LMICs. Thus, the focus of this study was to compare the SEPs for medicines used for three different cancer treatments (breast, prostate and colorectal) and its affordability and consequent impoverishment.

### Objectives

The main objectives of the study were to answer the following questions:

- Is the private sector purchasing medicines efficiently; what is the price of originator brand and generic medicines in the private sectors?
- What is the difference in price of the higher unit price and lowest unit price of the same cancer medicine?
- How affordable are medicines for the treatment of cancer for people with low income?

## Methods

The methodology employed in this study was adapted from the WHO/HAI methodology of measuring medicine prices and affordability [18] for ten cancer medicines (both originator brand (OB) and lowest priced generic (LPG) products) [2, 19]. The prices were based on the low, high and median 2020 SEP unit price per vial of the injectable cancer medicine formulation obtained from the South African Medicine Price Registry as of 11th March 2020 [19]. The South African Medicines Price Registry is managed by the National Department of Health and is a publicly available database that contains the current SEP prices of all registered medicines in South Africa, though previous versions of the database are available at fixed points in time. The database is an implementation of the transparent pricing policies for the private sector that is part of the South African legislation. All manufacturers are obliged to submit their SEPs to the National Department of Health, which are then entered into the database and published as an EXCEL spreadsheet on the website [19], which is continually updated as prices change. All the various prices (including if only one price was found) of each of the 10 medicines was extracted as submitted by the manufacturers to the database and included in the analysis [19]. Treatment regimens for advanced stages of Colo-rectal, breast and prostate cancer (being the most common amongst South African men and women [2] were taken from the Electronic Medicines Compendium (EMC) [20], United Kingdom (UK) and the National Comprehensive Cancer Network (NCCN) treatment guidelines [21].

A standardized computerized workbook [18] was used to enter and analyse data from the private sector on the components of medicine prices and the affordability of the medicines. The workbook for data entry automatically generates summary tables, which shows the median prices of the medicines. In this current study, the Median Price Ratio (MPR) was not calculated due to the outdated 2015 Management Sciences for Health (MSH), External Reference Price (ERP) [22]. Therefore, a comparison with the International Price Ratio (IPR) was not done. However, the median price unit was presented in individual medicines. The following research findings will be discussed:

- Procurement efficiency/brand premium which examines whether procurement prices are comparable amongst other types/brands of the same medicine. Medicine price variations between product types of the same medicine’s highest- and lowest-priced product, as well as between the OB and LPG, whereby analysis is limited to those medicines for which both product types were found (matched pair analysis). The difference is expressed in this paper as a ratio and a percentage.
- Cancer medicine affordability: affordability of those medicines for cancer usage based on treatment affordability in relation to the daily wage of the unskilled LPGW (WHO/HAI) [18] and the Niens et al. method [9, 17, 23].

Procurement efficiency is defined as the difference between the Highest-Priced Medicine (HPM) and Lowest-Priced Medicine (LPM) and brand premiums between the highest-priced generic or innovator brand products and their lowest-priced generic equivalents was determined [18]. The median SEP unit price was calculated rather than mean values. The percentage cost differential or price variation was calculated as;

$$ Cost\ Differential\ \left(\%\right)=\left[\left( Price\ of\ the\ Originator- Price\ of\ the\ generic\right)/ Price\ of\ Originator\right]\times 100. $$

The Price ratio between OB and LPG or HPM and LPM was calculated as*:*

$$ Price\ ratio= Price\ of\ the\  OB/ Price\ of\ the\  LPG\  or\ Price\ of\ the\  HPM/ Price\ of\ the\  LPM. $$

The maximum and the minimum of each medicine with the same strength regardless of it being OB or LPG was used to calculate the cost differential between minimum and maximum SEP (%).

For this study, medicine affordability has been investigated in terms of the days’ wages that a country’s unskilled LPGW needs to spend on a standard course of treatment [18]. This study presents patient prices and product affordability based on the WHO/HAI method [18] by examining the costs of cancer treatments and comparing them with the daily wage of the LPGW [18]. The 2020 salary of the unskilled LPGW in South Africa was 166.08 ZAR per day based on a 20.76 ZAR per hour and 8 h per day work schedule [24], which is equivalent to 9.9271 USD (1 USD = 16.73 ZAR, 12 September 2020) [25]. A month of oncology treatment was used to demonstrate the economic implication on a patient if they would have to pay for it out of pocket, even though a cancer patient is expected to have more than one cycle of treatment with multiple medicine regimens.

$$ Number\ of\ days\ wage\ to\ afford\ treatment= cost\ of\ vial(s)\  of\ cancer\ medicine\ needed\  per\  month/ daily\ wage\ of\ lowest\ paid\ government\ worker. $$

It is important to bear in mind that these costs refer only to the medicine component of the total treatment costs. Consultation fees and diagnostic tests may mean that the total cost to the patient is considerably higher. A limitation in the methodology of this study is the exclusion of the accompanying cost factors that play a role in the final cost to the cancer patient such as dispensing fees, facility fees, administration fees, doctors’ fees etc.

An additional measure of unaffordability using Niens et al. method [9, 17, 23] was included in this study. The unaffordability of a medicine also refers to the percentage of the population that is already below or would fall below the poverty line when having to procure the medicine [7–9, 17, 23]. We also used the impoverishment method to compare the proportion of the population below the poverty line (PL) before (I_pre_) and after (I_post_) the hypothetical procurement of a medicine [17]. For the percentage of the population represented by I_post_, the medicine is deemed unaffordable. Three types of data were required: medicine prices, aggregated income data (Y) [26], and information on the income distribution [17, 26]. (Ref: Table 1). We use the PL threshold of 5.50 USD [7, 8] or ZAR 92.02 [25] a day.

**Table 1:** Income distribution and average daily Income Per Capita (IPC) [26]

Cumulative % of population	Income group	Income distribution (%)	Average daily IPC ($)	Average daily IPC (ZAR)
*D*_1_ 0–10	Poorest 10%	0.9	0.89	14.91
*D*_2_ 10–20	Second poorest 10%	1.5	1.49	24.85
*D*_3_ 20–40	Second 20%	4.8	2.38	39.77
*D*_4_ 40–60	Third 20%	8.2	4.06	67.94
*D*_5_ 60–80	Fourth 20%	16.5	8.17	136.70
*D*_6_ 80–90	Second richest 10%	17.7	17.53	293.29
*D*_7_ 90–100	Richest 10%	50.5	50.02	836.78

South Africa Population = 58,558,270 and Household final expenditure (Y) =211,692,57 million US$

This study therefore focused solely on the SEP of the chosen cancer medicines for the most common cancer conditions in the private sector and how these affected the affordability. This study through its comparisons of OB to LPG sought to emphasis the cost savings implications of using the LPG in the treatment of a cancer patient.

## Results

The variation in the price of 10 cancer medicines of different strengths and dosage form was assessed (Table 2). The cost/price differential for 90% of all the medicines analysed was above 50%. The maximum variation was found in Doxorubicin 50 mg injection (97.33%), whereas Oxaliplatin 100 mg injection showed the minimum price variation (25.46%). when analysing the cancer medicines individually, Doxorubicin 50 mg injection had its highest priced medicine (37.44 times more expensive that its lowest priced medicine). Otherwise, almost all the cancer medicines (90%) in this analysis had significant price differences between their lowest and highest priced medicines with a cost differential ratio above 2.

**Table 2:** Comparison of Lowest Price with Highest Price of the same medicine (SEP)

No	Medicine Name	Medicine Strength	Dosage Form	Target Pack Size	Type of Cancer	Minimum SEP (ZAR)	Maximum SEP (ZAR)	Cost differential between Min and Max SEP (%)	Price Ratio	Number of generic Medicines	Number of Branded Medicines
1	Paclitaxel	300 mg	vial	1	Breast	24.8286	183.2814	86.45	7.38	9	1
2	Doxorubicin	10 mg	vial	1	Breast	16.2022	126.7600	87.22	7.82	5	2
3	Doxorubicin	50 mg	vial	1	Breast	16.2022	606.5330	97.33	37.44	8	2
4	Docetaxel	20 mg	vial	1	Breast/Prostrate	209.3080	789.7929	73.50	3.77	6	0
5	Docetaxel	80 mg	vial	1	Breast/Prostrate	279.0797	1490.2191	81.27	5.34	6	1
6	Fluorouracil	500 mg	vial	1	Colo-rectal	4.3900	36.4800	87.97	8.31	2	1
7	Oxaliplatin	50 mg	vial	1	Colo-rectal	41.6963	111.8818	62.73	2.68	3	0
8	Oxaliplatin	100 mg	vial	1	Colo-rectal	83.3926	111.8818	25.46	1.34	3	0
9	Irinotecan	40 mg	vial	1	Colo-rectal	211.9561	599.5800	64.65	2.83	1	1
10	Irinotecan	100 mg	vial	1	Colo-rectal	211.9529	708.0000	70.06	3.34	1	1

Table 3 shows the median price variability / cost differences between the OB and the LPG. Only those medicines for which both the originator brand and a generically equivalent product were found, were included in the analysis to allow for the comparison of prices between the two product types. Docetaxel 20 mg, Oxaliplatin 50 mg and Oxaliplatin 100 mg were excluded from the results as they had no OB for comparison. Results show that in the private sector, OBs cost more, on average, than their generic equivalents. The MPR ranged from 3.58–0.13, with 86% of the cancer medicines having an MPR above 1. The price variability between the OB and LPG for 66.7% of the medicines analysed was over 50%, this means that when OB medicines are prescribed/dispensed in the private sector, patients pay over 50% more than they would for generics. The highest cost differential was seen in Doxorubicin 100 mg (72.09%) followed by Irinotecan 100 mg (70.06%), Irinotecan 40 mg (64.65%) and Docetaxel 80 mg (62.13%) respectively. Thus, patients are paying substantially more to purchase OB medicines when LPGs are available.

**Table 3:** Price variation among different brands of cancer medicines available in private pharmacies database of South Africa

No	Medicine Name	Medicine Strength	Dosage Form (per unit)	Target Pack Size	Type of Cancer^a^	Median SEP Price per unit OB (ZAR)	Median SEP Price per unit LPG (ZAR)	Median Price Variation/Cost Differential (%)	Median Price Ratios
1	Paclitaxel	300 mg	vial	1	Breast	35.4918	27.5465	22.39	1.29
2	Doxorubicin	10 mg	vial	1	Breast	72.9250	20.3500	72.09	3.58
3	Doxorubicin	50 mg	vial	1	Breast	315.5469	213.4550	32.35	1.48
4	Docetaxel	80 mg	vial	1	Breast/Prostrate	1490.2191	564.2920	62.13	2.64
5	Fluorouracil	500 mg	vial	1	Colo-rectal	4.3900	34.2300	−679.73	0.13
6	Irinotecan	40 mg	vial	1	Colo-rectal	599.5800	211.9561	64.65	2.83
7	Irinotecan	100 mg	vial	1	Colo-rectal	708.0000	211.9529	70.06	3.34

^a^Regimens taken from the EMC and NCCN treatment guidelines [20, 21]

Price variability of the 28,6% surveyed OB and LPG cancer medicines had low-cost differentials less than 50%. The lowest was paclitaxel 300 mg (22.39%) followed by Doxorubicin 50 mg (32.35) respectively. The fluorouracil generic medicine was more expensive than its branded medicine, thus having a negative price variation of − 679.73%.

**Fig. 1:**
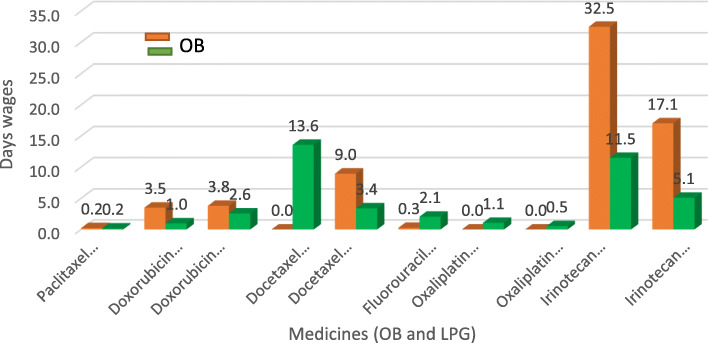
shows the affordability data for the selected cancer medicines

Affordability [18] (Ref: Tables 4 & 5 and Fig. 1) has been assessed only for 17 versions of OB and LPG cancer medicines from the private sector database. All OB treatments except for paclitaxel 300 mg (0.2 days wage) and Fluorouracil (Fluroblastin) 500 mg (0.3 days wage) costs respectively were more than 1 day’s wage. Among all the surveyed medicines, the OB of a one-month treatment of Irinotecan (Campto) 40 mg required 32.3 days’ wages and is the most unaffordable. The cost of generic versions of Irinotecan 40 mg was 11.5 days’ wages. For Docetaxel 80 mg the cost in days’ wages for the OB product is 9, while the LPG product cost is 3.4 days’ wages. For Irinotecan 100 mg the cost in days’ wages for the OB product is 17.1 while the LPG product cost is 5.1 days’ wages. For Doxorubicin 50 mg the cost in days’ wages for the OB product is 3.8 while the LPG product cost is 2.6 days’ wages. The cost of a one-month treatment with Doxorubicin 10 mg required about 3.5 days’ wages for the OB and 1-day wage for the LPG. For the LPG medicines without a comparator OB, buying Docetaxel 20 mg, Oxaliplatin 100 mg and Oxaliplatin 50 mg cost in days’ wages 13.6, 1.1 and 0.5 respectively. Moreover, Paclitaxel 300 mg OB, paclitaxel 300 mg LPG, Doxorubicin 10 mg LPG, Fluorouracil (Fluroblastin) 500 mg OB, Oxaliplatin 50 mg LPG and Oxaliplatin 100 LPG were found to be the most affordable cancer medicines in the private sector in South Africa. It is important to bear in mind that these costs refer only to the medicine component of the total treatment costs. Consultation fees and diagnostic tests may mean that the total cost to the patient is considerably higher.

**Table 4:** Treatment Regimen for calculating affordability [20]

Medicine	Strength	Dosage	Treatment Regimen per month
Paclitaxel	300 mg	220 mg/m2 once	1 vial (first Line treatment)
Doxorubicin	10 mg	75 mg/m2 once	8 vials
Doxorubicin	50 mg	75 mg/m2 once	2 vials
Docetaxel	20 mg	75 mg/m2 once	4 vials
Docetaxel	80 mg	75 mg/m2 once	1 vial
Fluorouracil	500 mg	15 mg/kg every week	10 vials (based on an 80 kg adult)
Oxaliplatin	50 mg	85 mg/m2 twice every month	2 vials
Oxaliplatin	100 mg	85 mg/m2 twice every month	1 vial
Irinotecan (Campto)	40 mg	350 mg/m2 once	9 vials
Irinotecan (Campto)	100 mg	350 mg/m2 once	4 vials

**Table 5:** Affordability in terms of the HAI method using number of day’s wages of a government worker required to pay for treatment with cancer medicine(s) [18]

No.	Medicine Name	Medicine Strength	Dosage Form	Target Pack Size	Medicine Type	SEP Median Price (ZAR)	Treatment (Number of vials needed per month)	Treatment Cost per month (ZAR)	Daily Wage (ZAR) (18)	Affordability (14)
1	Paclitaxel (Taxol)	300 mg	vial	1	OB	35.4918	1	35.4918	166.0800	0.2
2	Paclitaxel	300 mg	vial	1	LPG	27.5465	1	27.5465	166.0800	0.2
3	Doxorubicin (Adriblastina RD)	10 mg	vial	1	OB	72.9250	8	583.3996	166.0800	3.5
4	Doxorubicin	10 mg	vial	1	LPG	20.3500	8	162.8000	166.0800	1.0
5	Doxorubicin (Adriblastina CSV)	50 mg	vial	1	OB	315.5469	2	631.0938	166.0800	3.8
6	Doxorubicin	50 mg	vial	1	LPG	213.4550	2	426.9100	166.0800	2.6
7	Docetaxel	20 mg	vial	1	LPG	564.2920	4	2257.1680	166.0800	13.6
8	Docetaxel (Taxotere)	80 mg	vial	1	OB	1490.2191	1	1490.2191	166.0800	9.0
9	Docetaxel	80 mg	vial	1	LPG	564.2920	1	564.2920	166.0800	3.4
10	Fluorouracil (Fluroblastin)	500 mg	vial	1	OB	4.3900	10	43.9000	166.0800	0.3
11	Fluorouracil	500 mg	vial	1	LPG	34.2300	10	342.3000	166.0800	2.1
12	Oxaliplatin	50 mg	vial	1	LPG	89.0000	2	178.0000	166.0800	1.1
13	Oxaliplatin	100 mg	vial	1	LPG	89.0000	1	89.0000	166.0800	0.5
14	Irinotecan (Campto)	40 mg	vial	1	OB	599.5800	9	5396.2200	166.0800	32.5
15	Irinotecan	40 mg	vial	1	LPG	211.9561	9	1907.6045	166.0800	11.5
16	Irinotecan (Campto)	100 mg	vial	1	OB	708.0000	4	2832.0000	166.0800	17.1
17	Irinotecan	100 mg	vial	1	LPG	211.9529	4	847.8115	166.0800	5.1

Using the Niens et al. method [9, 17, 23], the proportion of the population living below the poverty line before (*I*_pre_) the hypothetical procurement of a medicine is 57%. The proportion impoverished after (*I*_post_) the hypothetical procurement of a medicine ranges up to 26%, making the most expensive medicine, Irinotecan (Campto) 40 mg OB, unaffordable to 82.95%. The proportion impoverished range from 0.3 to 17.8% for the rest of the medicines (Ref: Table 6) below.

**Table 6:** Medicine prices, cost of treatment per month and proportion impoverishment data [26]

No.	Medicine Name	Medicine Strength	Dosage Form	Target Pack Size	Medicine Type	SEP Median Price (ZAR)	Treatment (Number of vials needed per month)	Treatment Cost per month (ZAR)	I (post) %	The proportion impoverished I (post) − I (pre) %
1	Paclitaxel (Taxol)	300 mg	vial	1	OB	35.49	1	35.4918	57.35	0.35
2	Paclitaxel	300 mg	vial	1	LPG	27.55	1	27.5465	57.27	0.27
3	Doxorubicin (Adriblastina RD)	10 mg	vial	1	OB	72.93	8	583.3996	62.66	5.66
4	Doxorubicin	10 mg	vial	1	LPG	20.35	8	162.8000	58.58	1.58
5	Doxorubicin (Adriblastina CSV)	50 mg	vial	1	OB	315.55	2	631.0938	63.12	6.12
6	Doxorubicin	50 mg	vial	1	LPG	213.46	2	426.9100	61.14	4.14
7	Docetaxel	20 mg	vial	1	LPG	564.29	4	2257.1680	72.93	15.93
8	Docetaxel (Taxotere)	80 mg	vial	1	OB	1490.22	1	1490.2191	70.48	13.48
9	Docetaxel	80 mg	vial	1	LPG	564.29	1	564.2920	62.47	5.47
10	Fluorouracil (Fluroblastin)	500 mg	vial	1	OB	4.39	10	43.9000	57.43	0.43
11	Fluorouracil	500 mg	vial	1	LPG	34.23	10	342.3000	60.32	3.32
12	Oxaliplatin	50 mg	vial	1	LPG	89.00	2	178.0000	58.73	1.73
13	Oxaliplatin	100 mg	vial	1	LPG	89.00	1	89.0000	57.86	0.86
14	Irinotecan (Campto)	40 mg	vial	1	OB	599.58	9	5396.2200	82.95	25.95
15	Irinotecan	40 mg	vial	1	LPG	211.96	9	1907.6045	71.81	14.81
16	Irinotecan (Campto)	100 mg	vial	1	OB	708.00	4	2832.0000	74.76	17.76
17	Irinotecan	100 mg	vial	1	LPG	211.95	4	847.8115	65.22	8.22

I (pre) = 57%

## Discussion

People living with cancer’s survival depends on factors such as availability, affordability, and accessibility to treatment. Access to high-cost cancer medicines has become a major challenge in many countries, because of scarcity of pricing or affordability data to develop effective and transparent pricing policy, lack of insurance coverage and the resulting financially unaffordable cost to patients [11]. The health and economic objective of the South Africa NDP is to ensure the availability and accessibility of essential medicines and lower the cost of medicines in both the private and public sectors to all citizens [11, 13].

This study sought to analyse price variation among different brands of cancer medicines from the private sector and explore if the objectives of the NDP are being met with regards to oncology medicines [13]. The results of this study suggest that oncology medicine prices in South Africa are still high, and there are large price differences in the private sector between highest-priced and their lowest-priced equivalents, as well as between OB and LPG. The differences in price between HPM and LPM equivalents were found to be as high as 37.44 times in some instances. The private sector showed higher prices of OBs costing more than the LPGs with some having a cost difference of about 72.05%. Similar findings were also seen in studies conducted in LMICs (India, Nepal; and the African, Latin America, South East Asia, Western Pacific, East Mediterranean regions) on pricing of cancer medicines [3, 27–31]. These studies showed wide variations in price across different countries in regions, in the same country across different brands of the same medicine in the same dose and dosage form, individual medications and OBs versus LPGs [2, 27–32]. High patient prices can be due to lack of generic competition, suppliers of generic medicines pricing popular products only slightly below the originator brand version, high manufacturer profit margins, high government taxes and duties on medicines, and inefficient supply system.

Current pricing policies (or the lack thereof) have led to considerable variability in the prices of cancer medicines within a country [4]. There are various reasons for the observed price variations such as patent protection, monopolistic markets for new entities, regulatory issues, tax and tariffs, geographic location, income status and lack of internal price regulation measures. In LMICs, improving health system strengthening is the key and it can improve various facets of medicine chain including access and affordability of medicines [33]. Differences in guidelines of medicine regulating authorities of various countries and their pricing policies account for the varying prices of medicines among different countries [27].

Assessing the prices of chemotherapy medicines in the private sector showed that the price differences between the OB and the LPG for the medicines used in this study ranged from the OB being 1.29 to 3.58 times more than the price of the LPG. In this study, Fluorouracil 500 mg’s LPG was more expensive than its OB with a negative cost differential of 679.73%, which may be because of generic competition or some other factors. For paclitaxel, ten LPG cancer medicines were available with only one OB. These results indicate that there are savings that can be achieved by using the LPGs. The need for the LPG to be available and improve the affordability of cancer medicines was highlighted. About 33% of the medicines have a ratio of almost 1 between OBs and LPGs, which suggests that the SEP policy may be hindering competition of some products by setting a price ceiling or capping increases. Alternatively, companies may be using the OB price as a guide to their price setting. The existence of generics on the market does affect originator prices in some countries. In some countries, originator prices might have decreased because of generic competition, whereas in other countries originator prices remained at a high level [31]. South Africa has a large and highly developed private pharmaceutical manufacturing system and market estimated to account for 25% of the volume but 65% of the market by value [11, 31]. Generic medicines were estimated to account for about 65% of all items dispensed in the private sector and 40% of expenditure [34]. In South Africa, an area of importance has been that of generic penetration, in response to the legal requirements for mandatory offer of generic substitution by all dispensers [12].

As shown by this study, large differences in the price of the same medicine (by a different manufacturer) might affect patients’ expenditure on medicines, especially when the patient does not know about price variation and cheaper alternatives, or if a medical scheme has included a different medicine on their formulary. The patient may still face high co-payments as part of their plan with a medical scheme. In terms of South African private sector pricing, the SEP mechanism and the publication of annual adjustments has provided some transparency in medicines pricing in terms of the full medicines price (without the dispensing fee addition). Medical schemes should follow suit and publish their co-payment schedule on their website to allow patients to understand the costs they incur, and to determine if alternatives exist where no co-payment is required. The government should continue in its efforts to promote generic prescribing and utilization, generic substitution, transparent pricing, efficient regulation, empower patients to r request cheaper alternatives, improve price transparency by medical schemes, introduce internal and external reference pricing benchmarking, health technology assessment processes and apply pharmacoeconomic analyses to negotiate the SEP prices of cancer medicines [11, 12, 17, 35].

Pricing policies will also have to be reviewed based on the fluctuations in South African currency, which could impact on the supply of essential cancer medicines. The regulated maximum SEP mechanism, with annual adjustments, may need reconsideration and refinement, and its increases need to consider exceptional circumstances that may arise as the result of extreme currency fluctuations within a given calendar year [11, 12].

Regular revision of the national medicines policy which confronts the demands of a purchaser-provider split and a completely reformed health financing system are needed to guide pharmaceutical practice in the future. Such a policy will need to build on the gains achieved [11].

In treating the commonly occurring cancer conditions in South Africa using standard regiments, affordability of generics seems to be an issue in the Irinotecan 40 mg, Irinotecan 100 mg, Doxorubicin 50 mg, Docetaxel 20 mg and Fluorouracil 500 mg. The LPGW would need between 0.2–13.6 days’ wages to purchase lowest priced generic medicines from the private sector. If OBs are prescribed/dispensed, costs escalate to between 0.2–3 2.5 days’ wages, respectively. The LPG could be up to 67% more affordable than the OB. Some treatments were clearly unaffordable, e.g. the treatment of Colorectal cancer with OB or LPG Irinotecan (Campto) 40 mg would cost 32.5 days’ or 11.5 wages respectively. Thus, for some cancer medicines, a month’s wage would not be enough to afford treatment.

Affordability data indicated that 57% of the population would not be able to pay for their cancer medicines as they live below the poverty line before (*I*_pre_) the hypothetical procurement of the cancer medicines. Irinotecan (Campto) 40 mg OB is expensive and was unaffordable to 82.95% of the population. Our findings were consistent with Niens et al. study, on the impoverishing effects of purchasing medicines using the measure of the proportion of a population that fell below a relevant poverty line after buying medicines which concluded that the impoverishing effect of medicines varied between OB and the LPG products and that a substantial portion of the population would be pushed into poverty as a result of medicine procurement [9]. Another study showed that the monthly costs of biological cancer medicines in Pakistan were higher than 20% of the monthly household income after spending on food [36]. Only 58.1% of non-biological cancer medicines were affordable [36].

The private sector cancer medicines are funded largely from insurance premiums (paid by individuals and employers) but also from out-of-pocket payments [11]. In South Africa, only 17.1% of people belonged to a medical scheme in 2017 [10]. Thus if these medicines are not covered by health insurance, the unaffordable prices will prevent these medicines from being used for a substantial portion of cancer patients and impact their health if they had to pay for their treatment out-of-pocket or if these medicines were unavailable in the public sector.

The results of our findings are consistent with a study [37] which showed that the affordability of LPGs (67.9%) cancer medicines was more as compared to OBs (53.4%) in the private sector in Pakistan. Studies in India and Bangladesh, on affordability of paediatric cancer medicines revealed that cancer treatments were not affordable for most families leading to treatment abandonment [31, 38].

A study comparing prices in Australia, China, India, Israel, South Africa, the United Kingdom, and the United States, linked the price of cancer medicines to affordability using international markers of wealth and showed that there were major differences in patterns of affordability between countries [39]. Medicines in South Africa were less affordable than in all high-income countries, including the US where prices were considerably higher. These differences were driven by lower levels of wealth in middle-income countries. In understanding differences in wealth between countries there may be some debate regarding the most appropriate metric to use, as GDP per capita does not incorporate personal income that may be impacted by unemployment levels, retirement age, and social patterns of employment. Differential pricing may be an acceptable policy to ensure global affordability of highly active cancer therapies.

High inflation, low per capita income and increasing cost of living are among the several hurdles that hinders people from affording cancer medication. Differential pricing, low premium insurance schemes, medicine discounts, patient-access schemes, tax benefits, concerted public-private initiatives, patent changes, national health plans emulation of salient models in governance and public health administration is required for long term sustainability [31, 38, 40]. The relationship between price and healthcare outcomes should be enhanced through arrangements that reward innovation, while ensuring the sustainability of an affordable healthcare system [38, 41, 42].

### Limitations of the research

The medicines included in this study was from the private sector database, and thus there may be concern that the research is not representative of the situation in South Africa.. This study, using basic indicators only, cannot give a complete picture of the pharmaceutical sector in South Africa. The median price ratio was not calculated, and therefore, the data collected were not comparable with the international reference prices. Results on affordability may also lead to over-estimation since the calculation used was based on the lowest-paid government workers’ wages. A significant proportion of the population earn less than the LPGW. The calculation of affordability utilizes the standard dose of individual medicines and affordability may vary if patients are taking more than one medicine.

## Conclusions and recommendations

Cancer is expensive to treat. The results of the study show that the affordability and price of medicines in South Africa is of concern. As the country moves towards National Health Insurance, options for patients incurring high-cost treatment needs to be considered and formulated. The Government of South Africa has regulated the prices of medicines; however, more needs to be done and further strategies are needed to address the high costs of cancer medicines. This requires multi-faceted interventions, as well as the review and refocusing of policies, regulations and educational interventions. A recommendation for future research would be to investigate the impact of medicine price bench marking for oncology medicines in the private sector of South Africa.

## Data Availability

The data and materials are available on request from the authors.

## Abbreviations

EMC: Electronic medicines compendium
ERP: External Reference Price
GDP: Gross Domestic Product
GNI: Gross National Income
HAI: Health Action International
HER2 +: Human Epidermal growth factor Receptor 2 positive
HICs: High-Income Countries
HPM: Highest-Priced Medicine
IPC: Income Per Capita
Ipost: Income post-payment
Ipre: Income pre-payment
IPR: International Price Ratio
LMIC: Lower Middle-Income Country
LPG: Lowest Priced Generic
LPGW: Lowest Paid Government Worker
LPM: Lowest-Priced Medicine
MPR: Median Price Ratio
MSH: Management Sciences for Health
NCCN: National Comprehensive Cancer Network
NDP: National Drug Policy
OB: Originator Brand
OOP: Out-of-pocket
PL: Poverty Line
SEP: Single Exit Price
UK: United Kingdom
UMIC: Upper Middle-Income Country
US: United States
WB: World Bank
WHO: World Health Organization
ZAR: South African Rand
